# Thermal fault diagnosis of complex electrical equipment based on infrared image recognition

**DOI:** 10.1038/s41598-024-56142-x

**Published:** 2024-03-06

**Authors:** Zongbu Tang, Xuan Jian

**Affiliations:** 1 Infrastructure Department, Shanghai University of Traditional Chinese Medicine, Shanghai, 201203 China; 2 Power Supply Service Command Center, State Grid Beibei Power Supply Company, Chongqing, 400070 China

**Keywords:** Complex electrical equipment, Thermal fault diagnosis, Infrared image, Temperature difference, Semantic segmentation, Refined detection, Computer science, Information technology, Scientific data

## Abstract

This paper realizes infrared image denoising, recognition, and semantic segmentation for complex electrical equipment and proposes a thermal fault diagnosis method that incorporates temperature differences. We introduce a deformable convolution module into the Denoising Convolutional Neural Network (DeDn-CNN) and propose an image denoising algorithm based on this improved network. By replacing Gaussian wrap-around filtering with anisotropic diffusion filtering, we suggest an image enhancement algorithm that employs Weighted Guided Filtering (WGF) with an enhanced Single-Scale Retinex (Ani-SSR) technique to prevent strong edge halos. Furthermore, we propose a refined detection algorithm for electrical equipment that builds upon an improved RetinaNet. This algorithm incorporates a rotating rectangular frame and an attention module, addressing the challenge of precise detection in scenarios where electrical equipment is densely arranged or tilted. We also introduce a thermal fault diagnosis approach that combines temperature differences with DeeplabV3 + semantic segmentation. The improved RetinaNet's recognition results are fed into the DeeplabV3 + model to further segment structures prone to thermal faults. The accuracy of component recognition in this paper achieved 87.23%, 86.54%, and 90.91%, with respective false alarm rates of 7.50%, 8.20%, and 7.89%. We propose a comprehensive method spanning from preprocessing through target recognition to thermal fault diagnosis for infrared images of complex electrical equipment, providing practical insights and robust solutions for future automation of electrical equipment inspections.

## Introduction

Substations serve as fundamental units within the power system, primarily responsible for the reception, transformation, and distribution of electric energy. They house critical electrical equipment, including potential transformers, current transformers, circuit breakers, and switches^1^. The collective functioning and stable operation of this equipment are pivotal for ensuring the safety and reliability of power transmission. Most electrical equipment in substations is exposed to the outdoor environment, which subjects it to long-term degradation from harsh weather conditions, foreign object intrusion, frequent operation, and other factors, leading to rust, blockages, insulation degradation, or even equipment failure^2,3^. Statistically, failures in essential electrical equipment, such as transformers and switches, are frequently characterized by abnormal heating phenomenon, including the corrosion of the switches, poor contact of the circuit breakers, deterioration, and moisture of potential transformers, etc.^4^. Prompt and accurate detection of abnormal temperatures is vital for assessing the operational status of electrical equipment, playing a crucial role in maintaining the safety and stability of substations^5^.

The acquisition of temperature information for substation electrical equipment largely depends on infrared thermography (IRT). Thanks to its non-contact nature, extensive temperature measurement range, and high efficiency, IRT is extensively employed in routine inspections, particularly for detecting temperatures in electrical equipment^6,7^. IRT operates by using sensors to measure the target's thermal radiation power, which, after photoelectric conversion and signal processing and other means of processing, results in outputting a thermal spectrum that maps the temperature distribution of the equipment. This allows for early detection of abnormal temperature distributions, enabling timely maintenance or replacement to prevent accident escalation^8^. Presently, operators continue to use handheld infrared thermal imagers for manual temperature recording or install them near significant power equipment for continuous monitoring^9^.

The daily inspection of power equipment generates a massive amount of infrared images. It remains necessary to manually assess whether the equipment exhibits temperature abnormalities^10^. This method, only suitable for analyzing and diagnosing a limited number of image tasks, cannot cope with the detection of a large volume of infrared images. Moreover, the reliance of the human eye judgment on the experience of professionals may lead to fatigue, potentially resulting in diagnostic errors^11^. Additionally, the often low resolution of infrared images further complicates manual analysis^12^. Consequently, it is essential to develop automatic analysis algorithms for infrared images to ensure the reliable diagnosis of thermal faults in electrical equipment and to enhance the intelligence level of the power system.

A novel infrared image denoising algorithm for electrical equipment based on DeDn-CNN is proposed. This algorithm introduces a deformable convolution module that autonomously learns the noise feature information in infrared images. An image enhancement method utilizing Weighted Guided Filtering (WGF) with an Anisotropic Single-Scale Retinex (Ani-SSR) is also proposed, which replaces Gaussian wrap-around filtering with anisotropic diffusion filtering to mitigate the issue of strong edge halos. The RetinaNet is augmented by incorporating a rotating rectangular frame and an attention module, and further enhanced by appending the Path Aggregation Network (PAN) to the Feature Pyramid Network (FPN) for improved bottom-up feature fusion. A thermal fault diagnosis method for electrical equipment based on the DeeplabV3 + semantic segmentation model is introduced, which leverages temperature differences for fault determination. This study proposes a comprehensive method ranging from preprocessing to recognition to thermal fault diagnosis of infrared images, offering practical insights and robust solutions for automating the infrared inspection of electrical equipment.

## Infrared image preprocessing

### Image denoising

**Figure 1:**
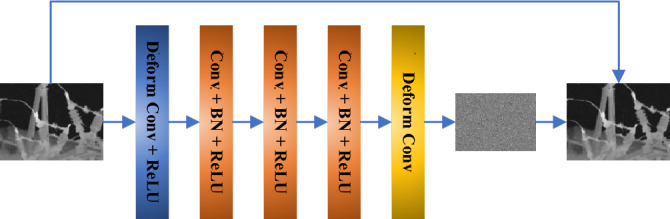
Structure of the DeDn-CNN.

Image denoising involves processing degraded images that contain noise to estimate the original image. Traditional Denoising Convolutional Neural Networks (Dn-CNN) use a fixed 3 × 3 convolutional kernel for noise feature extraction in images. However, Dn-CNN mainly learns noise information from images containing noise, without accommodating shape rules, which limits the effectiveness of feature extraction with a fixed-shape convolutional kernel^13^. To overcome this, a deformable convolution module is introduced to enhance the DeDn-CNN, which employs a deformable 3 × 3 convolution in place of the original convolution operation. The network's first layer is modified from Conv + ReLU to Deform Conv + ReLU, and the last layer is changed from Conv to Deform Conv, as depicted in Fig. 1.

**Figure 2:**
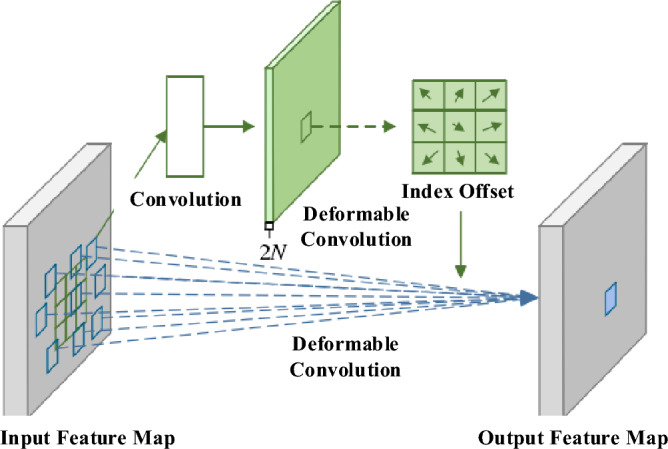
Diagram of deformable convolution.

The deformable convolution module introduces an offset to the sampling points, as illustrated in Fig. 2. The top part generates the index offset by processing the input feature map through a regular convolution layer, while the bottom part convolves the input feature map with the corresponding kernel to produce the output feature map^14^. The deformable convolution kernels are capable of adapting to the extraction of complex noise patterns in images.

### Image enhancement

The original infrared image is decomposed into two layers—basic and detail—using Weighted Guided Filtering (WGF). These layers are processed individually and then combined to produce the enhanced image. For the basic layer, which suffers from low contrast and poor quality, an improved SSR algorithm integrated with anisotropic diffusion filtering is employed to adjust the grayscale, enhancing dark regions in the image and improving overall contrast. For the detail layer, which contains numerous edge and texture features, an arctan nonlinear function is applied to emphasize these details without introducing additional noise.

#### Image layering based on weighted guided filtering

Traditional guided filtering applies a fixed regularization factor *ε* to each region of the image, which does not take into account the textural differences among various regions. To address this limitation, WGF introduces an edge weighting factor Γ_*G*_, allowing *ε* to be adaptively adjusted based on the degree of image smoothing, thereby enhancing the algorithm's capability to preserve image edges^15^. The edge weighting factor Γ_*G*_ and the modified linear factor *a*_*k*_ are defined in the following equation.

$$ \Gamma_{G} (i) = \frac{1}{N}\sum\limits_{i = 1}^{N} {\frac{{\delta_{G,i}^{2} (i) + \gamma_{\sigma } }}{{\delta_{G,i}^{2} \left( {i^{\prime } } \right) + \gamma_{\sigma } }}} ,\quad a_{k} = \frac{{\delta_{k}^{2} }}{{\delta_{k}^{2} + \varepsilon /\Gamma_{G} (i)}} $$

where ${\delta }_{G,i}^{2}(i)$ is the variance within the window *w*_*k*_ of the image centered on pixel *i*, Γ_*G*_(*i*) is the use of the current window variance divided by the variance of all the windows in the whole image and then take the mean, *N* is the number of all the pixels, and *L* is the distribution range of the image grayscale level^16^.

If the pixel is situated in a region of the image with sharp variations, the variance within the window centered around the pixel will be larger, causing the Γ_*G*_(*i*) to be greater than 1. This increase leads to a higher value of *a*_*k*_, which in turn better preserves edge details. In contrast, in smoother regions of the image, the Γ_*G*_(*i*) will likely be less than 1, resulting in a decrease in *a*_*k*_ and a smoother output in the filtered image.

WGF is employed to process the input image, yielding a smoother base layer, and the detail layer image is obtained by subtracting this base layer from the original image, as illustrated in the following equations^17^.

$$ q = WGF(p), \, O = p - q $$

where *p* is the original image to be enhanced, *q* is the output basic layer after weighted guided filtering, *O* is the decomposed detail layer, and *WGF* is the operation of weighted guided filtering. The basic layer image is subsequently augmented by the improved SSR algorithm for subsequent enhancement. The detail layer *O* is processed by a nonlinear function to suppress the noise information in the image, and the expression is shown:

$$ E_{o} (x,y) = \arctan [O(x,y)] $$

#### Ani-SSR algorithm

According to Retinex theory, the illumination component of an image is relatively uniform and changes gradually. Single-Scale Retinex (SSR) typically uses Gaussian wrap-around filtering to extract low-frequency information from the original image as an approximation of the illumination component *L*(*x*, *y*). However, Gaussian wrap-around filtering tends to skew the estimate of the illumination component at the strong edges of the image, often resulting in a pronounced halo effect around object edges in the enhanced image^18^. As a solution, anisotropic diffusion filtering is utilized in place of Gaussian wrap-around filtering. This alternative approach provides a more accurate estimation of the illumination at image boundaries and reduces halo artifacts at strong edges. The anisotropic diffusion equation is presented below.

$$ \frac{\partial A}{{\partial t}} = div\left[ {c(x,y,t)\nabla A} \right] = \nabla c\nabla A + c(x,y,t)\Delta A $$

where *A* is the input grayscale image; *t* is the diffusion time; *div* is the dispersion operator; $\nabla$ is the partial derivative i.e. gradient operator; Δ is the Laplace operator; *c* is the diffusion function, which controls the diffusion.

$$ \begin{gathered} c(x,y) = g\left( {\left\| {\nabla A} \right\|} \right) \\ g\left( {\left\| {\nabla A} \right\|} \right) = \frac{1}{{1 + \left( {{{\nabla A} \mathord{\left/ {\vphantom {{\nabla A} k}} \right. \kern-0pt} k}} \right)^{2} }} \\ \end{gathered} $$

where *k* is the thermal conductivity coefficient, which controls the filtering sensitivity, the larger the value of *k* the smoother the image obtained, but at the same time the image details will become blurred^19^. $\Vert \bullet \Vert$ is the norm for calculating the difference between predicted noise and true noise. Anisotropic diffusion filtering is used instead of Gaussian wrap-around filtering, which makes the estimation of the light component at the image boundary more accurate, and attenuates the halo at the strong edge part of the enhanced image.

### Preprocessing results

**Figure 3:**
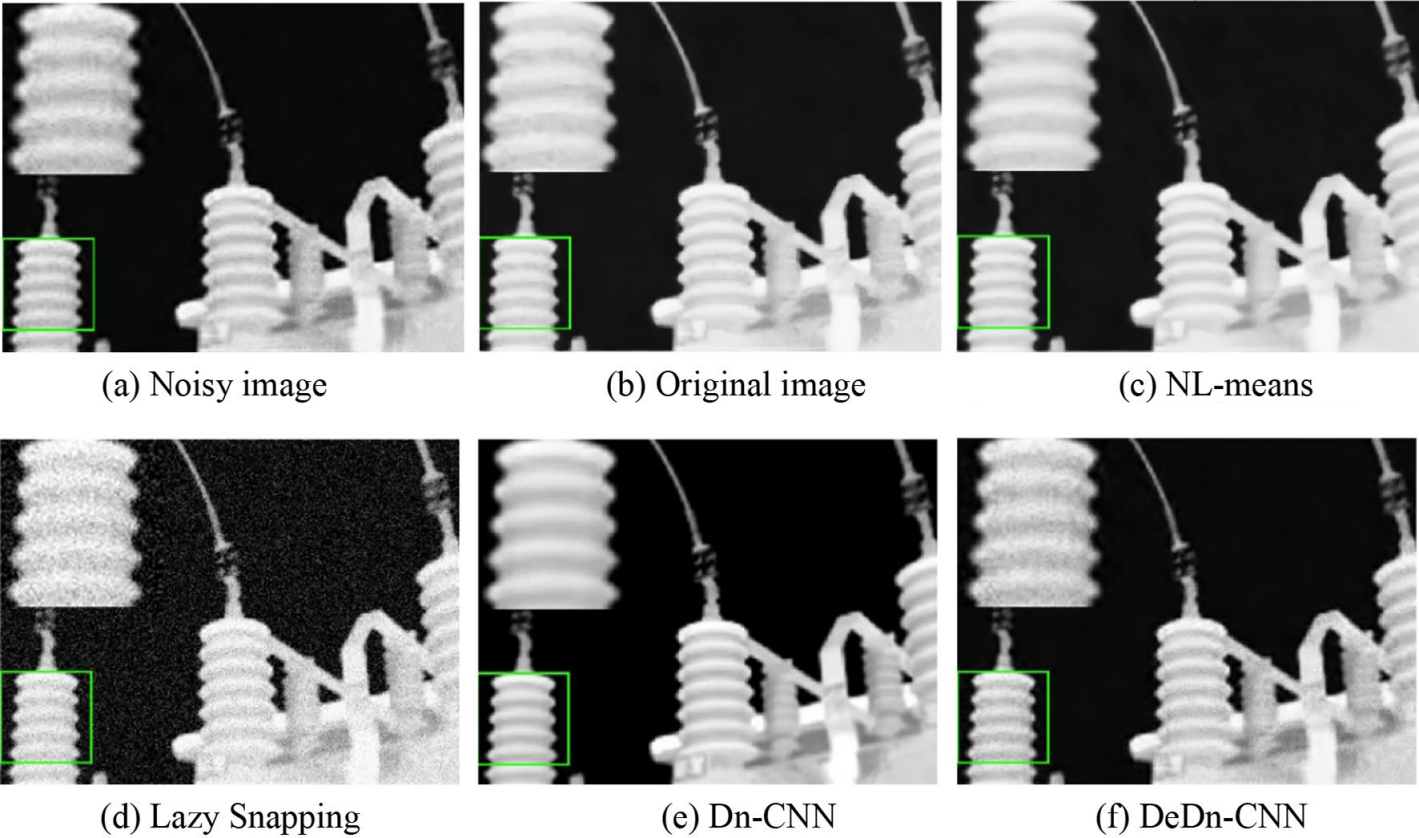
Comparison of image denoising.

Infrared temperature measurements were conducted using a Testo 875-1i thermal imaging camera at various substations in Northwest China. A total of 508 infrared images of complex electrical equipment, each with a pixel size of 320 × 240, were collected. Out of these, 457 were randomly selected as the training set after artificial noise was added, and the remaining 51 images formed the test set. The DeDn-CNN was benchmarked against the Dn-CNN, NL-means^20^, wavelet transform^21^, and Lazy Snapping^22^ for denoising purposes, as shown in Fig. 3.

An analysis of Fig. 3 reveals that the NL-means and wavelet transform denoising effects are somewhat inferior compared to Dn-CNN, with more residual noise remaining after NL-means processing and more severe image distortion. The infrared image denoised with Dn-CNN has fewer residual noise spots because Dn-CNN autonomously extracts more abstract feature information from the noise by learning the difference between the noise map and the clean map, rather than relying on manually summarized statistical noise properties. This allows it to better fit the noise distribution of the image. The DeDn-CNN achieves superior denoising results as it is better adapted to noise with chaotic distributions and irregular shapes during feature extraction, leaving the least amount of noise in the image post-denoising and attaining higher image fidelity. The average PSNR for NL-means, wavelet transform, Dn-CNN, and DeDn-CNN are 33.47, 34.82, 38.25, and 40.33, respectively, which further demonstrates that DeDn-CNN is more effective at removing noise from infrared images.

**Figure 4:**
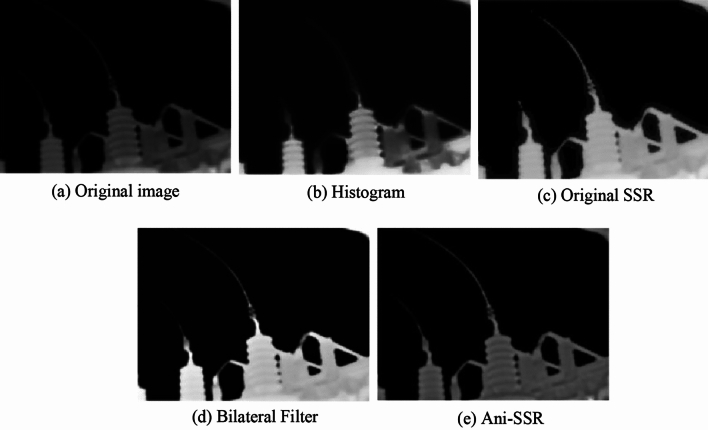
Comparison of image enhancement results.

The Ani-SSR algorithm is compared with histogram equalization, the original SSR, and the bilateral filter layering^23^, as depicted in Fig. 4. The original infrared image exhibits a low overall gray level, low contrast, and a suboptimal visual effect. Histogram equalization enhances the brightness and contrast of the image but results in a diminished range of gray levels and more significant degradation of image details. The original SSR enhancement of the infrared image leads to a pronounced halo effect, and a serious loss of texture, which hinders subsequent equipment recognition. The results from the bilateral filter indicate an issue of over-enhancement, causing the image to be overexposed and visually unappealing. In contrast, Ani-SSR successfully improves image contrast while preserving rich edge information and texture details. It overcomes the problem of halo effects in the original SSR, particularly at strong edges with drastic gradient changes, and provides superior overall enhancement of the infrared image of electrical equipment.

The average gradient (AG) is also used as an evaluation index for assessment, as shown in equation.

$$ AG = \frac{1}{mn}\sum\limits_{i = 1}^{m} {\sum\limits_{j = 1}^{n} {G_{ij} } } $$

where *G*_*i*,*j*_ is the gradient value of the pixel at (*i*, *j*) in the image. The larger the AG, the richer the information of edge texture is represented, and the comparison of AG of each algorithm is shown in Table 1. From Table 1, it is evident that the original SSR achieves a lower Average Gradient (AG) due to its inability to adapt to regions with drastic edge changes, as it utilizes a Gaussian function during the enhancement process, resulting in the loss of image edges and texture details. The Ani-SSR, by preserving more image details while enhancing contrast, exhibits an improvement in the average gradient score compared to the other three algorithms, objectively demonstrating the effectiveness of the proposed algorithm in this paper.

**Table 1:** Comparison of AG score.

	AG
Histogram	1.42
Original SSR	1.33
Bilateral Filter	1.59
Ani-SSR	1.88

## Refined detection of complex electrical equipment

The single-stage target detection network, RetinaNet^24,25^, has been improved to better suit the detection of electrical equipment, which often has a large aspect ratio, a tilt angle, and is densely arranged. The horizontal rectangular frame of the original RetinaNet has been altered to a rotating rectangular frame to accommodate the prediction of the tilt angle of the electrical equipment. Additionally, the Path Aggregation Network (PAN) module and an Attention module have been incorporated into the feature fusion stage of the original RetinaNet.

### Original RetinaNet

Contemporary mainstream target detection networks fall into two categories: two-stage target detection algorithms exemplified by Faster-RCNN and one-stage target detection algorithms such as the YOLO algorithms. The former relies on a Region Proposal Network (RPN), which introduces additional computational complexity, while the latter directly predicts the target classification confidence and location parameters through regression computation, typically with lower accuracy. RetinaNet employs the Focal Loss function to balance the weights of difficult and easy samples within the loss calculation, merging the benefits of both detection accuracy and speed^26^.

**Figure 5:**
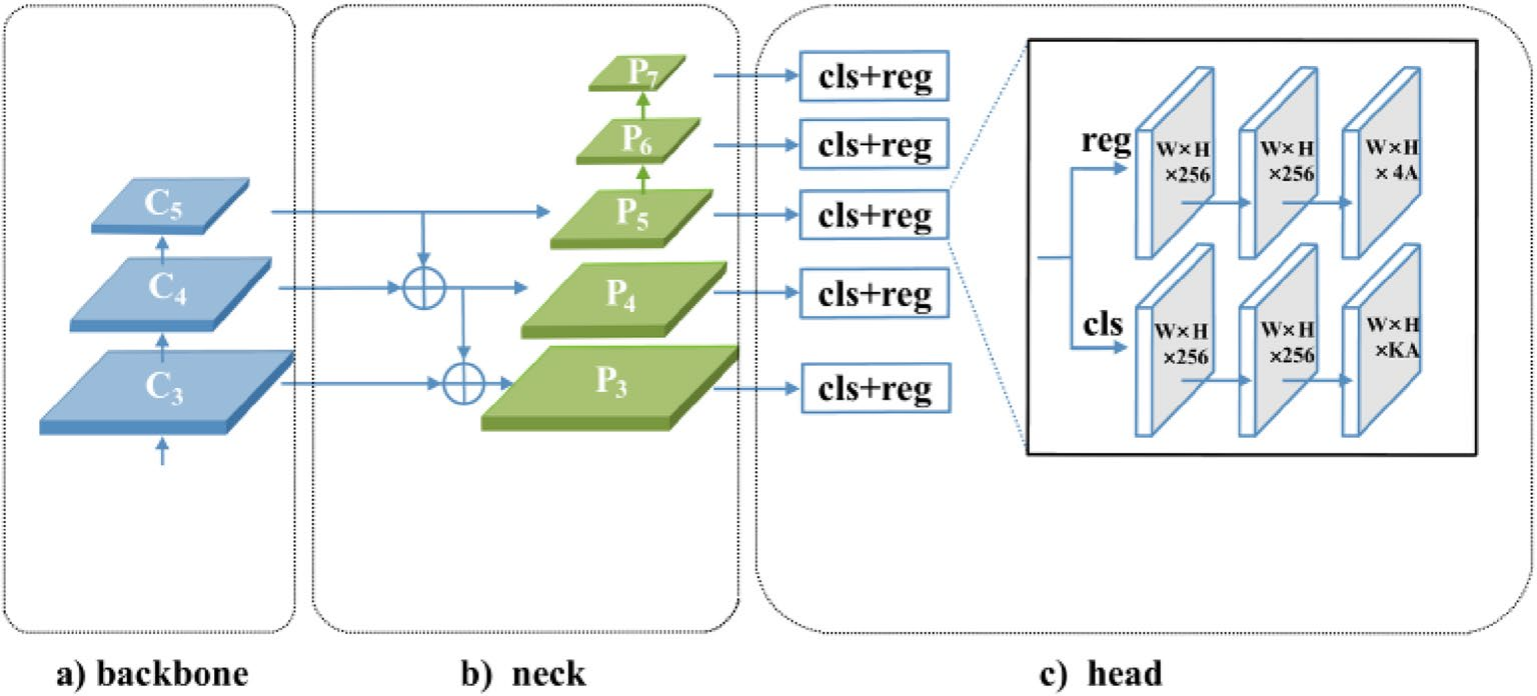
RetinaNet structure.

RetinaNet comprises three components: the backbone, neck, and head, as illustrated in Fig. 5. The backbone is primarily responsible for feature extraction, often utilizing ResNet-101; the neck uses Feature Pyramid Networks (FPN), which integrates features from different scales outputted by the backbone to adapt to objects of various sizes; the head, employing Fully Convolutional Networks (FCN), predicts the target location regression parameters and classification confidence for different scale feature maps^27^.

### Improving RetinaNet

#### Rotating rectangular frame

**Figure 6:**
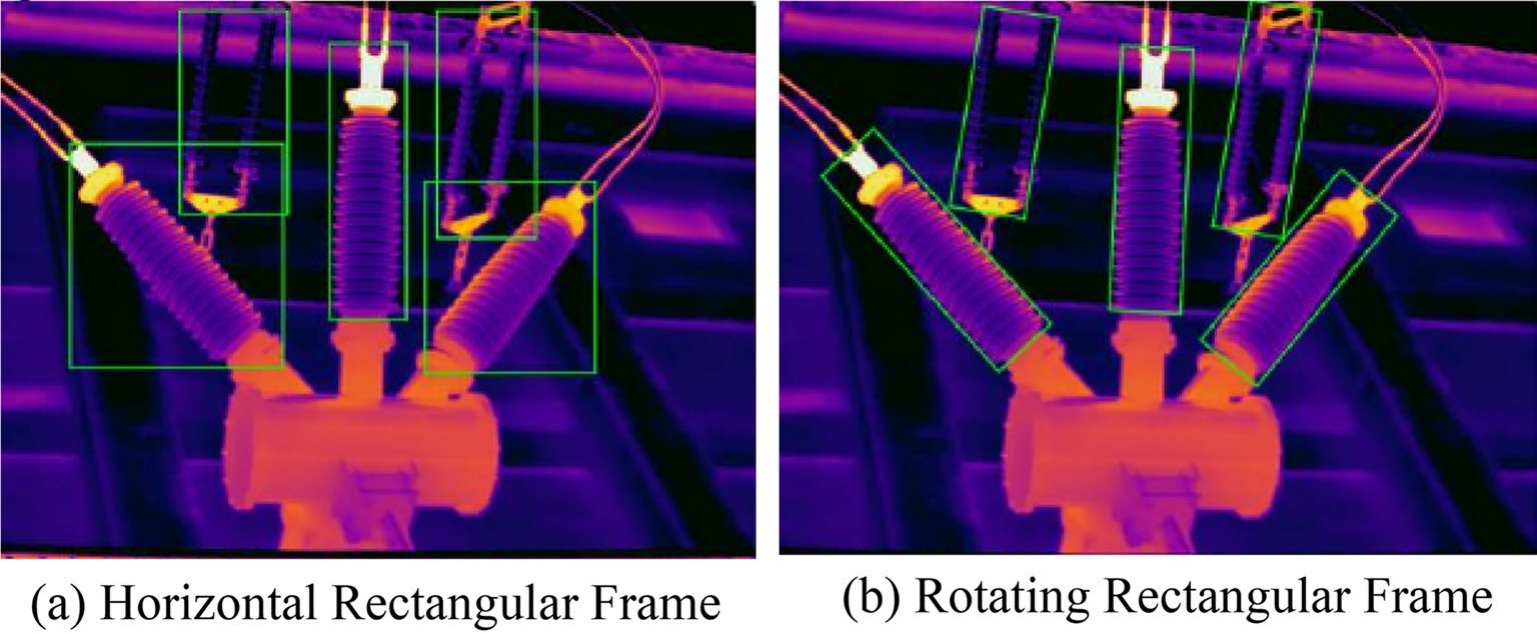
Comparison of the detection effect of two frames.

Given the dense arrangement and potential tilt of electrical equipment due to the angle of capture, the standard horizontal rectangular frame of RetinaNet may only provide an approximate equipment location and can lead to overlaps. When the tilt angle is significant, such as close to 45°, the horizontal frame includes more irrelevant background information. By incorporating the prediction of the equipment's tilt angle and modifying the horizontal rectangular frame to a rectangular frame with a rotation, the accuracy of localization and identification of electrical equipment can be considerably enhanced. The comparison results of the two detection frames are displayed in Fig. 6.

**Figure 7:**
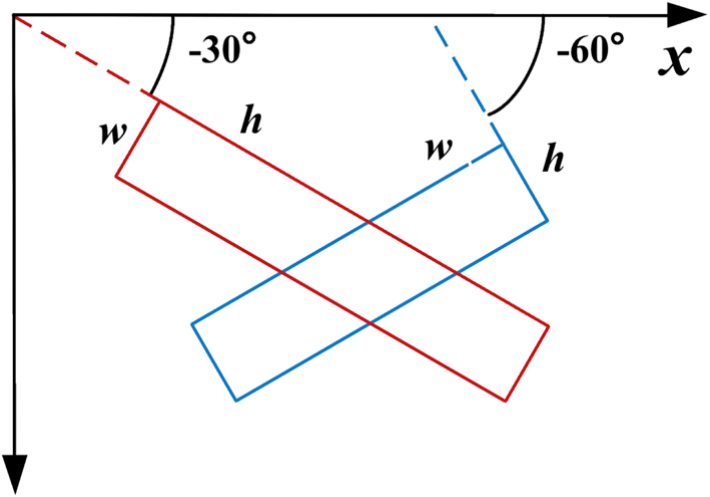
Diagram of rotating rectangular frame.

The rotational frame defined in this paper is illustrated in Fig. 7. Here, the side forming an acute angle with the positive direction of the *x*-axis is labeled as *h*, while the other side of the rectangle is identified as *w*. The angle is defined as the acute angle between *h* and the *x*-axis, with its value ranging from [−π / 2,0). To define a frame with a rotation, five parameters are necessary: (*x*, *y*, *w*, *h*, *θ*), which represent the coordinates, width, height, and inclination angle, respectively.

**Figure 8:**
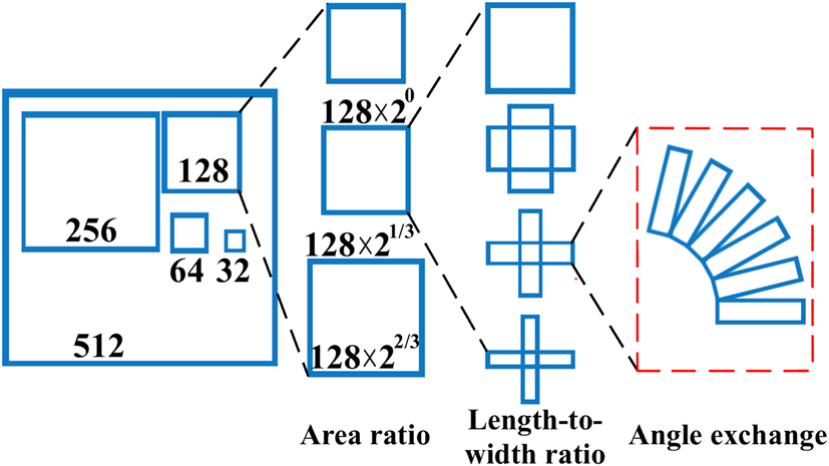
Improved mechanism.

The pixel area at five different detection scales are 32^2^, 64^2^, 128^2^, 256^2^, and 512^2^,. Each pixel area includes three scale factors of [2^0^, 2^1/3^, 2^2/3^] and three aspect ratios of [0.5, 1, 2], resulting in the creation of nine frames. Since electrical equipment typically have elongated shapes with large aspect ratios, this paper extends the original three aspect ratio factors to seven scales: [1:1, 1:2, 2:1, 1:3, 3:1, 1:5, 5:1]. This modification improves adaptability to the elongated shapes of electrical equipment in infrared images. Regarding the rotation angle, six transformation factors of [− *π* / 2, − 5*π* / 12, − *π* / 3, − *π* / 4, − *π* / 6, − *π* / 12 ] are introduced, increasing the number of original horizontal rectangular frames from 9 to 126, as depicted in Fig. 8.

#### Attention mechanism

**Figure 9:**
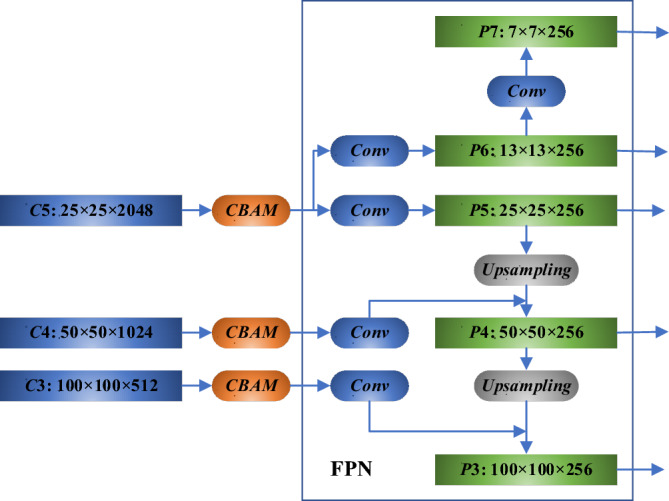
FPN structure by adding attention module.

The Attention module enhances the network's capability to discern prominent features in both the channel and spatial dimensions of the feature map by integrating average and maximum pooling. In this paper, the detection target is power equipment in substations, environments that are often cluttered and have complex backgrounds. Therefore, the network is improved with the Attention module^28^. The addition of the Attention module to the shallow layer feature maps does not significantly enhance performance due to the limited number of channels and the minimal feature information extracted at these levels. Conversely, implementing it in the deeper network layers is less effective since the feature map's information extraction and fusion operations are already complete; it would also unnecessarily complicate the network. Consequently, in this study, the Attention module is introduced after the backbone and before the FPN module, as shown in Fig. 9.

#### Path aggregation network (PAN)

**Figure 10:**
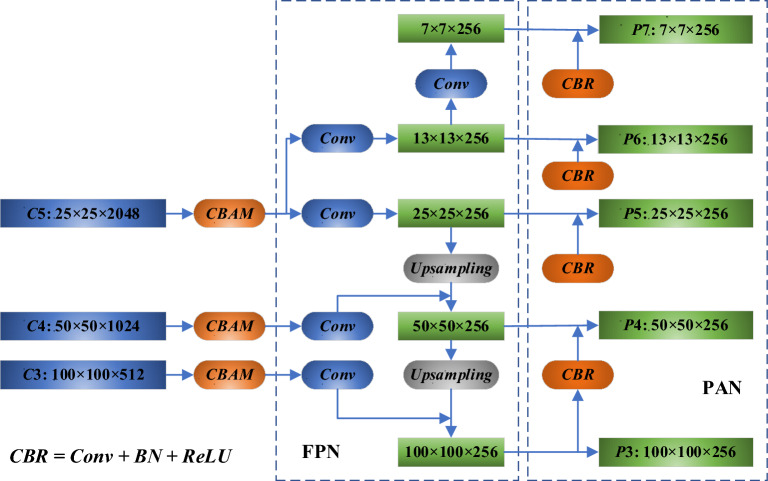
FPN structure with attention and PAN.

The Path Aggregation Network (PAN) is incorporated subsequent to the FPN module, as indicated in Fig. 10. The original FPN module conveys the deep feature map's strong semantic information to the shallow feature map via a "top-down" approach but does not carry the detailed target location and texture information from the shallow feature map to the deep feature map^29^. The PAN structure enables a "bottom-up" feature fusion mechanism by downsampling the shallow feature map with Conv + BN + ReLU and then superimposing it onto the deeper feature map. This approach enriches the target texture and position information conveyed from the shallow to the deeper feature map. The integration of the FPN and PAN modules optimizes the use of features extracted by the backbone, fuses feature parameters across different layers, and addresses the limitation of single-scale feature maps in one-stage methods, which may not effectively represent object location and semantic information across multiple scales simultaneously.

#### Head structure and loss function

**Figure 11:**
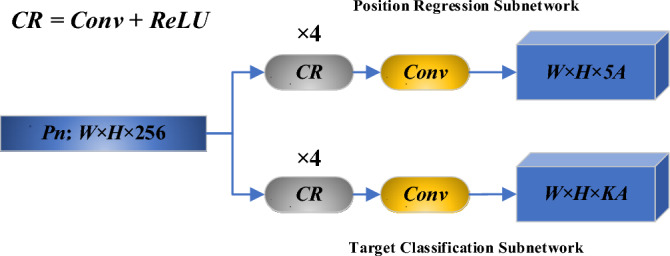
Improved FCN of RetinaNet.

The original head predicts the classification confidence parameter and the location regression parameter using the Fully Convolutional Networks (FCN)^30^. due to the increase in the number of frames in this paper, it is necessary to change the FCN appropriately, as presented in Fig. 11. The original RetinaNet only needs to predict the 4 parameters $({t}_{x}{\prime},{t}_{y}{\prime},{t}_{w}{\prime},{t}_{h}{\prime})$ of the horizontal rectangular frame, so the last layer outputs the tensor of W × H × 4A. The rotating rectangular frame adds the prediction of the angular, such that it is imperative to adjust the network to predict the 5 parameters of $({t}_{x}{\prime},{t}_{y}{\prime},{t}_{w}{\prime},{t}_{h}{\prime},{t}_{\theta }{\prime})$, outputting the tensor of W × H × 5A, as illustrated in Fig. 11.

The loss function of the original RetinaNet is divided into two parts: classification loss and position regression loss. The electrical equipment with tilt angle is detected accurately, so the angular offset of the target should be added to the loss function of position regression, as shown in the following equation.

$$ \left\{ {\begin{array}{*{20}l} {t_{x} = \left( {x - x_{a} } \right)/w_{a} } \hfill \\ {t_{y} = \left( {y - y_{a} } \right)/h_{a} } \hfill \\ {t_{w} = \lg \left( {w/w_{a} } \right)} \hfill \\ {t_{h} = \lg \left( {h/h_{a} } \right)} \hfill \\ {t_{\theta } = \theta - \theta_{a} } \hfill \\ \end{array} } \right. $$

where (*x*, *y*, *w*, *h*, *θ*) and (*x*_*a*_, *y*_*a*_, *w*_*a*_, *h*_*a*_, *θ*_*a*_) are the position coordinates and tilt angle of the real frame and predicted frame, respectively, and (*t*_*x*_, *t*_*y*_, *t*_*w*_, *t*_*h*_, *t*_*θ*_) represents the offset of the predicted frame relative to the real frame. The loss value of position regression is calculated based on Smooth L1 function.

$$ SL_{1} \left( {t_{i} - t_{i}^{\prime } } \right) = \left\{ {\begin{array}{*{20}c} {0.5\left( {t_{i} - t_{i}^{\prime } } \right)^{2} } & {\left| {t_{i} - t_{i}^{\prime } } \right| < 1} \\ {\left| {t_{i} - t_{i}^{\prime } } \right| - 0.5} & { \, else \, } \\ \end{array} } \right. $$

where the value range of *t*_*i*_ is (*t*_*x*_, *t*_*y*_, *t*_*w*_, *t*_*h*_, *t*_*θ*_) and the value range of *t*_*i*_' is $({t}_{x}{\prime},{t}_{y}{\prime},{t}_{w}{\prime},{t}_{h}{\prime},{t}_{\theta }{\prime})$. The calculation of the total loss value of target classification and position regression is:

$$ L = \frac{{\lambda_{1} }}{N}\sum\limits_{n = 1}^{N} {t_{n}^{\prime } } \sum\limits_{{i \in \{ x,y,w,h,\theta \} }} S L_{1} \left( {t_{ni}^{\prime } ,t_{ni} } \right) + \frac{{\lambda_{2} }}{N}\sum\limits_{n = 1}^{N} {L_{cls} } \left( {p_{n} ,t_{n} } \right) $$

where *N* denotes the number of frames; ${t}_{n}{\prime}$ takes 1 when the frame is foreground, and 0 when the frame is background; ${t}_{ni}{\prime}$ represents the coordinate offset of the predicted position corresponding to the *n*-th frame; and ${t}_{ni}$ expresses the coordinate offset of the *n*-th frame with respect to the real frame; *p*_*n*_ denotes the value of the multicategory confidence distribution of the *n*-th frame predicted by the sub-network after the Sigmoid function is computed, and *t*_*n*_ expresses the belonging category label of the *n*-th frame corresponding to the real target. *L*_*cls*_ denotes the category loss, calculated using the Focal Loss function of the original RetinaNet; the parameters *λ*_1_ and *λ*_2_ are taken as 1 by default.

### Performance comparison

Infrared images of six types of substation equipment—insulator strings, potential transformers (PTs), current transformers (CTs), switches, circuit breakers, and transformer bushings—were selected for recognition. The detection accuracy of the improved RetinaNet is evaluated using Average Precision (AP) and mean Average Precision (mAP). AP assesses the detection accuracy for a specific type of electrical equipment, while mAP is the mean of the APs across all equipment types, indicating the overall detection accuracy. AP and mAP are defined as follows.

$$ \left\{ {\begin{array}{*{20}l} {P = \frac{TP}{{TP + FP}}} \hfill \\ {R = \frac{TP}{{TP + FN}}} \hfill \\ \end{array} } \right. $$

$$ AP = \int_{0}^{1} P (R)dR $$

$$mAP = \frac{{\sum\limits_{n = 1}^{6} {AP} (n)}}{6}$$

where *TP* represents the number of positive samples classified correctly, *FP* represents the number of negative samples incorrectly classified as positive samples, *FN* is the number of positive samples incorrectly labeled as negative samples, and *P* and *R* are the detection rate and accuracy rate, respectively.

**Figure 12:**
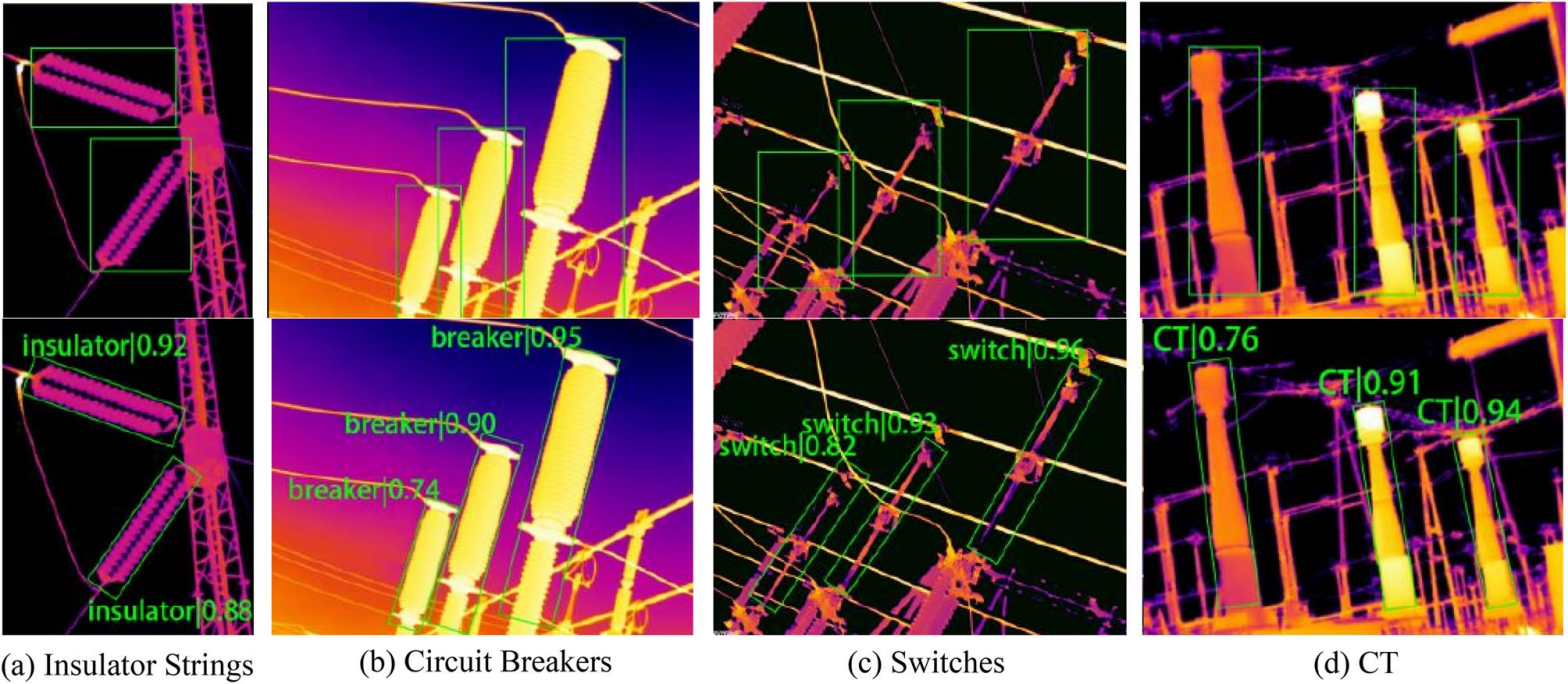
Comparison of detection results (original RetinaNet and improved RetinaNet).

Table 2 presents the APs and mAPs for different models detecting six types of electrical equipment, including Faster R-CNN, YOLOv3, the original RetinaNet, and the improved RetinaNet. The improved RetinaNet's AP values surpass those of the other three models for all six equipment types. The model's mAP is 1.9 percentage points higher than that of the original RetinaNet, indicating improved detection accuracy. Additionally, in scenarios where electrical equipment is densely arranged at various angles, the rotating rectangular frame achieves more precise detection than the horizontal frame, as illustrated in Fig. 12. A tilted electrical equipment's rotating rectangular frame introduces less background information than the horizontal rectangular frame, and there is less overlap in the detection results of the densely arranged electrical equipment,, aiding in the separation of the equipment for fault diagnosis based on thermal information.

**Table 2:** Comparison of detection results of different models.

Model	AP			PT	CT	Transformer Bushings	mAP
	Circuit Breakers	Insulator Strings	Switches				
Faster-RCNN^31^	95.3	89.8	87.7	97.4	96.3	94.6	93.5
YOLOv3^31^	92.5	87.3	83.0	92.5	91.5	91.2	89.7
Original RetinaNet^24^	95.8	90.8	89.2	96.9	95.4	92.2	93.4
Improved RetinaNet	96.4	93.8	90.9	98.8	97.0	95.0	95.3

Analyzing Fig. 12, we see that the two rows display the detection effects of the original RetinaNet and the improved RetinaNet, respectively. Figures 12a,b show that insulator strings and CTs, which have large tilt angles, are poorly served by algorithms using horizontal rectangular frames as these introduce a significant amount of irrelevant background images unrelated to the electrical equipment. In contrast, the improved RetinaNet more accurately contours the edges of the equipment, reducing the inclusion of extraneous background information. Figures 12c,d demonstrate that, due to the camera angle, the equipment appears not only tilted but also densely arranged, which challenges the traditional horizontal rectangular frame-based detection networks in separating individual equipment. The improved RetinaNet utilizes rotating frames to locate and identify equipment, circumventing the limitations of conventional framing and reducing overlap, thereby achieving more precise detection outcomes.

## Thermal fault diagnosis of complex electrical equipment

### Semantic segmentation of electrical equipment

**Figure 13:**
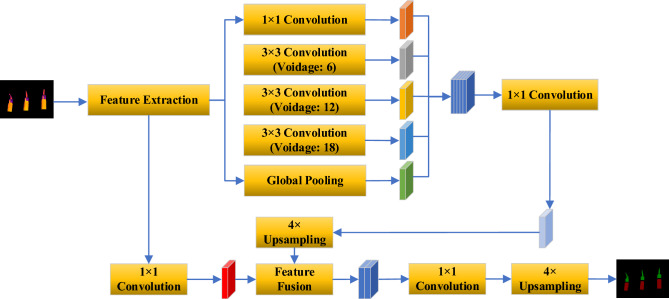
DeeplabV3 + structure.

Semantic segmentation involves the pixel-wise classification according to different semantics based on pixel features, as exemplified in Fig. 13. DeeplabV3 + utilizes a classic encoder-decoder structure^32^. Its encoder eliminates pooling operations to preserve more detail and positional information. Additionally, by incorporating a channel-separable convolution module, the encoder decouples spatial from channel information, reducing parameter count during network training^33^. The decoder produces prediction maps that match the original image's resolution—for instance, Fig. 13 classifies pixels on the top of the transformer bushing and the bushing itself^34^. Our focus is directed toward segmenting three vulnerable structures: the cap of the transformer bushing (Cap), the disconnecting link of switches (Disconnecting Link), and the potential transformer bushing (Bushing).

### Fault diagnosis of of thermal fault-prone structures

The relative temperature-difference method employs the temperature-difference information of the corresponding positional temperature values of two equipment with the same or similar basic states, such as category, load, and environment, to identify faults. Firstly, the temperature difference between the corresponding temperature points of two equipment is measured, then the temperature-rise value of the higher temperature point among the two points is calculated. Lastly, the relative temperature difference *δ*_*t*_ is computed using the ratio of the two, which is formulated in the following function:

$$ \delta_{t} = \frac{{\tau_{1} - \tau_{2} }}{{\tau_{1} }} \times 100\% = \frac{{\left( {T_{1} - T_{0} } \right) - \left( {T_{2} - T_{0} } \right)}}{{T_{1} - T_{0} }} \times 100\% = \frac{{T_{1} - T_{2} }}{{T_{1} - T_{0} }} \times 100\% $$

where *δ*_*t*_ is the relative temperature difference between the two equipment under test, *τ*_1_ is the temperature-rise of the hot spot under test (unit: K), *T*_1_ is the temperature of the hot spot (unit: K), *τ*_2_ and *T*_2_ are the temperature-rise and temperature of the normal temperature point, and *T*_0_ is the ambient temperature.

Relative temperature-difference method is primarily applicable to the current-heating faults judgment, especially for the abnormal heating caused by the small load current, the relative temperature-difference method can reduce the probability of leakage judgment of the small current load defect.

Similar comparison method refers to the same working condition, the same external environment of the same type of equipment temperature comparison to determine the equipment thermal defects, can be used for fault diagnosis of potential-heating faults.

Diagnostic criteria are set for Cap, Disconnecting Link, and Bushing. Cap and Disconnecting Link are prone to current-heating faults, are shown in Table 3. For Bushing, it is easy to have potential-heating faults. If the temperature difference is less than 2 K, it is determined that there is no faults, and if the temperature difference is greater than this threshold, it is determined that there is a potential-heating fault.

**Table 3:** Diagnostic criteria for faults.

Components	General faults	Severe faults	Critical faults
Disconnecting link	*δ*_*t*_ ≥ 35%; hot spot temperature < 90 ℃	*δ*_*t*_ ≥ 80%; Hot spot temperature 90 ℃ ~ 130 ℃	Hot spot temperature > 130 ℃; *δ*_*t*_ ≥ 95% and hot spot temperature > 90 ℃
Cap	*δ*_*t*_ ≥ 35%; hot spot temperature < 55 ℃	*δ*_*t*_ ≥ 80%; Hot spot temperature 55 ℃ ~ 80 ℃	Hot spot temperature > 80 ℃; *δ*_*t*_ ≥ 95% and hot spot temperature > 55 ℃

#### Thermal fault diagnosis of the cap

**Figure 14:**
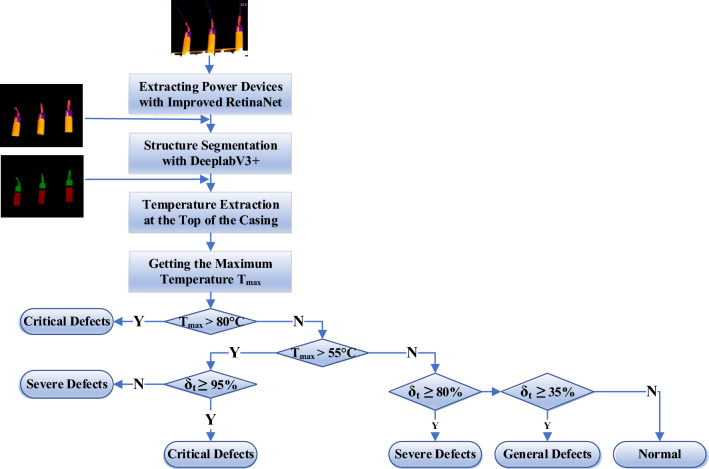
Diagnostic process of the cap.

The Cap is prone to current-heating faults, often due to internal bolt loosening or wiring aging corrosion and other reasons that increase the resistance, resulting in an increase in the amount of heat generated. Figure 14 illustrates the fault diagnosis process of the Cap. Initial detection of Cap is carried out using improved RetinaNet, and the results are input into DeeplabV3 + model for segmentation, thus separating *n* regions of the Cap. The local temperature maximum *T*_1_, *T*_2_, *T*_3_…*T*_*n*_ are yielded, the maximum value is selected as the hot spot temperature *T*_max_ and the minimum value is selected as the normal temperature *T*_min_, and the relative temperature difference *δ*_*t*_ is obtained. If the *T*_max_ and *δ*_*t*_ satisfy the discriminating conditions, it is determined as the corresponding fault level, and if they do not satisfy the conditions, it is judged that the equipment is normal.

#### Thermal fault diagnosis of the disconnecting link

**Figure 15:**
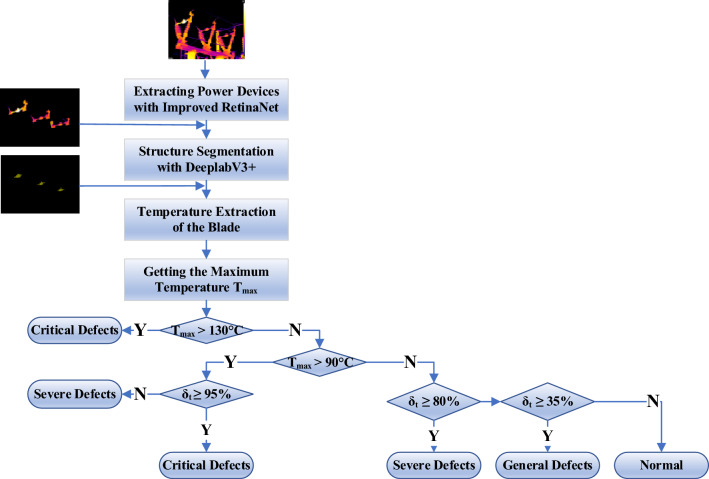
Diagnostic process of the disconnecting link.

The Disconnecting Link is prone to current-heating faults. Frequent reversing operations of the Disconnecting Link often result in insufficient spring clamping force of the contact fingers and abrasion of the contact fingers. Figure 15 illustrates the fault diagnosis process of the Disconnecting Link. The local temperature maximum *T*_1_, *T*_2_, *T*_3_…*T*_*n*_ are obtained, the maximum value is selected as the hot spot temperature *T*_max_ and the minimum value is selected as the normal temperature *T*_min_, and the relative temperature difference *δ*_*t*_ is obtained. The *T*_max_ and *δ*_*t*_ are adopted to determine whether the equipment is faulty.

#### Thermal fault diagnosis of the bushing

**Figure 16:**
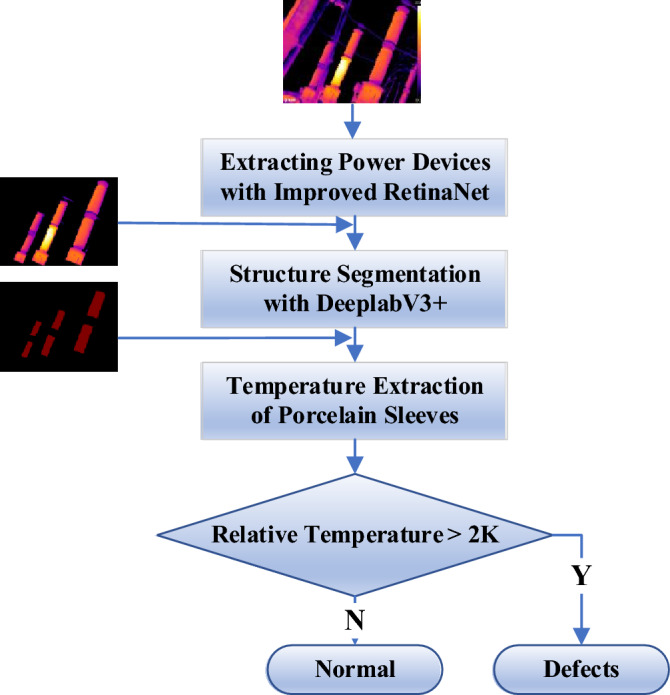
Diagnostic process of the bushing.

The Bushing is prone to abnormal heating due to the failure of the internal capacitance unit, and is a potential-heating fault. Capacitor unit fault primarily arises from moisture, capacitive components aging and other factors, usually in the wet season is more frequent. Fault diagnosis process of the Bushing is shown in Fig. 16. Since the Bushing belongs to the potential-heating fault, the basis for judgment differs from the current-heating fault. Initial detection of potential transformers was performed using improved RetinaNet, and the results were input into the DeeplabV3 + model for segmentation. The maximum temperatures *T*_1_, *T*_2_, *T*_3_…*T*_*n*_ were extracted for each region, and the hotspot temperature max(*T*_1_, *T*_2_, *T*_3_…*T*_*n*_) and the normal temperature min(*T*_1_, *T*_2_, *T*_3_…*T*_*n*_) were selected. If the temperature difference exceeds 2 K, it is determined that the Bushing has occurred a potential-heating fault; otherwise it is determined to be normal.

#### Experimental analysis

A selection of 282 infrared images containing bushings, disconnecting links, and PTs was chosen for fault diagnosis. The test set includes 47 infrared images of thermal faults on bushings and 52 images showing abnormal heating at disconnecting links, as shown in Table 4. The images of PTs comprise 44 with faults and 38 without faults. The fault diagnosis results for the three types of equipment are displayed in Tables 5, 6, and 7, respectively.

**Table 4:** Fault diagnosis data set.

Type	Fault level			
	Normal	General fault	Severe fault	Critical fault
Bushing	40	19	15	13
Disconnecting link	61	22	19	11

**Table 5:** Fault diagnosis results of the cap.

Sample	Diagnostic results			
	Normal	General fault	Severe fault	Critical fault
Normal sample	37	2	1	0
General fault	2	16	1	0
Severe fault	0	1	14	0
Critical fault	0	1	1	11

**Table 6:** Fault diagnosis results of the disconnecting link.

Sample	Diagnostic results			
	Normal	General fault	Severe fault	Critical fault
Normal sample	56	2	3	0
General fault	2	19	1	0
Severe fault	1	2	16	0
Critical fault	0	1	0	10

**Table 7:** Fault Diagnosis Results of the Bushing.

Sample	Diagnostic results	
	Normal	Fault
Normal sample	36	2
Fault sample	3	41

**Figure 17:**
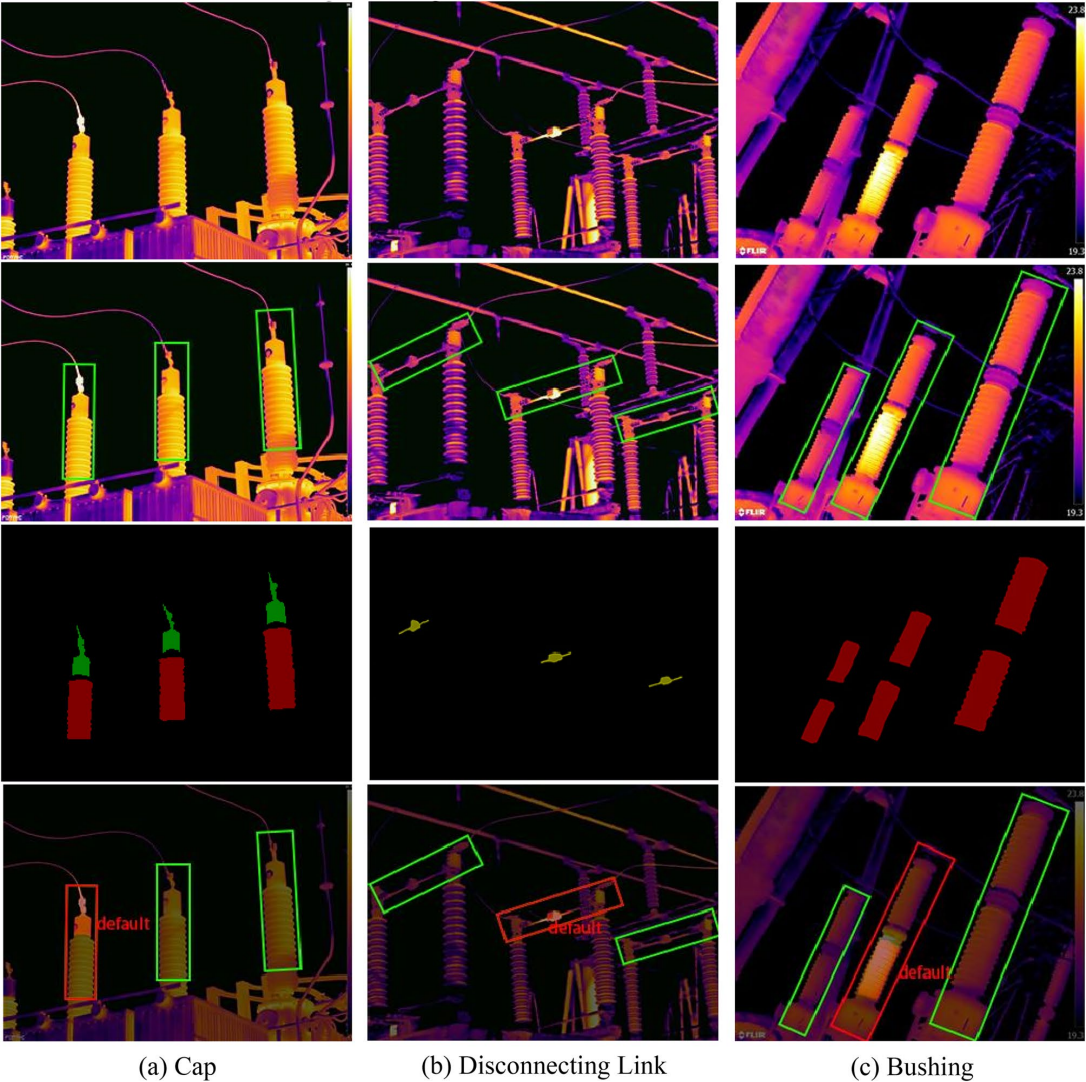
Diagnostic effect of some images.

Of the 143 fault images, faults were identified in 41 images of caps, 45 images of disconnecting links, and 40 images of PT bushings. The recognition accuracies reached 87.23%, 86.54%, and 90.91%, with false alarm rates of 7.50%, 8.20%, and 7.89%, respectively. The recognition results for some of the thermal fault images are presented in Fig. 17. The cap shown in Fig. 17 exhibits a current-induced heating fault due to corrosion. The maximum temperature of the cap was 59.5 °C, the normal temperature was 25.9 °C, and the relative temperature difference *δ*_*t*_ was 85.06%. The algorithm in this paper identifies this as a severe fault, which is consistent with the actual sample's fault level. The disconnecting link underwent oxidation due to long-term operational switching, causing an abnormal temperature rise. The maximum temperature recorded for the structure was 103.3℃, the normal temperature was 41.4℃, and the *δ*_*t*_ was 70%. The diagnostic model in this paper classified this as a severe fault. The temperature difference between the faulty and non-faulty states of the bushing was 3.2 K, exceeding the judgment threshold, indicating a potential heating fault.

## Conclusion

This paper presents a fault diagnosis method for electrical equipment based on deep learning, which effectively handles denoising, detection, recognition, and semantic segmentation of infrared images, combined with temperature difference information. A comprehensive approach is proposed, ranging from preprocessing to recognition, for diagnosing thermal faults in infrared images of electrical equipment. This contributes valuable experience and viable solutions for future automation of electrical equipment inspection.

1. A denoising algorithm for infrared images, DeDn-CNN, is introduced. It incorporates a deformable convolution module into the Dn-CNN to autonomously learn noise features in infrared images. Additionally, an image enhancement algorithm based on WGF and Ani-SSR is proposed, which employs anisotropic diffusion filtering instead of Gaussian wrap-around filtering, thus avoiding the issue of strong edge halos during image enhancement.
2. An improved electrical equipment detection algorithm based on RetinaNet is proposed. It utilizes rotating rectangular frames to enable refined detection in cases where electrical equipment is densely arranged or at an angle. An attention module is integrated to deal with the complex backgrounds typical of substations, and a PAN is appended after the FPN to achieve bottom-up feature map fusion.
3. A thermal fault diagnosis method is proposed that combines temperature difference information with DeeplabV3 + semantic segmentation. The enhanced RetinaNet recognition results are fed into the DeeplabV3 + model for further segmentation of thermal fault-prone structures, and fault diagnosis is performed by leveraging the temperature difference data.

## Data Availability

All data used in the paper can be obtained from the Zongbu Tang (corresponding author).
